# Biosynthetic Potential of *Hypogymnia* Holobionts: Insights into Secondary Metabolite Pathways

**DOI:** 10.3390/jof9050546

**Published:** 2023-05-09

**Authors:** Nadim Ahmad, Manfred Ritz, Anjuli Calchera, Jürgen Otte, Imke Schmitt, Thomas Brueck, Norbert Mehlmer

**Affiliations:** 1 Werner Siemens Chair of Synthetic Biotechnology, Department of Chemistry, Technical University of Munich (TUM), 85748 Garching, Germany; 2 Senckenberg Biodiversity and Climate Research Centre (SBiK-F), Senckenberganlage 25, 60325 Frankfurt am Main, Germany; 3 Institute of Ecology, Evolution and Diversity, Goethe University Frankfurt, Max-von-Laue-Straße 13, 60438 Frankfurt am Main, Germany

**Keywords:** *Hypogymnia physodes*, *Hypogymnia tubulosa*, long read sequencing, polyketide synthesis, biosynthetic gene cluster, lichen, reference genome

## Abstract

Lichens are symbiotic associations consisting of a photobiont (algae or cyanobacteria) and a mycobiont (fungus). They are known to produce a variety of unique secondary metabolites. To access this biosynthetic potential for biotechnological applications, deeper insights into the biosynthetic pathways and corresponding gene clusters are necessary. Here we provide a comprehensive view of the biosynthetic gene clusters of all organisms comprising a lichen thallus: fungi, green algae, and bacteria. We present two high-quality PacBio metagenomes, in which we identified a total of 460 biosynthetic gene clusters. Lichen mycobionts yielded 73–114 clusters, other lichen associated ascomycetes 8–40, green algae of the genus *Trebouxia* 14–19, and lichen-associated bacteria 101–105 clusters. The mycobionts contained mainly T1PKSs, followed by NRPSs, and terpenes; *Trebouxia* reads harbored mainly clusters linked to terpenes, followed by NRPSs and T3PKSs. Other lichen-associated ascomycetes and bacteria contained a mix of diverse biosynthetic gene clusters. In this study, we identified for the first time the biosynthetic gene clusters of entire lichen holobionts. The yet untapped biosynthetic potential of two species of the genus *Hypogymnia* is made accessible for further research.

## 1. Introduction

Lichens were historically regarded as a symbiotic organism comprising a fungus and photosynthetic partners (algae/cyanobacteria) [1]. However, more recent studies suggested that lichen individuals host not only the primary lichen-forming fungus (mycobiont) and primary photosynthetic partner (photobiont), but also additional fungi belonging to the Basidiomycota [2], or Ascomycota [3], as well as a diverse suite of bacteria and other algae [4,5]. Lichens have therefore been referred to as ecosystems [6], or holobionts [7]. Due to their unique secondary metabolite spectrum, these composite organisms have gained increasing attention over the past decades [8]. Lichen mycobionts synthesize a plethora of natural products [9]. The most prominent classes comprise depsides, depsidones, dibenzofurans, and phenolic compounds [10,11]. Confirmed bioactivities of these compounds encompass antimicrobial [8,12,13,14], antifungal [15], anti-inflammatory [16], antioxidant [17,18], and anticancer [14,19] functions. For the latter, medically relevant examples are physodic acid, gyrophoric acid, and atranorin [20]. An inhibitory effect on metabolic enzymes was also detected in evernic acid, physodic acid, usnic acid, and atranorin [10,21]. Bacteria associated with lichens have also been shown to possess diverse compounds with pharmaceutically promising activities [22,23,24].

To exploit these compounds biotechnologically, it is crucial to elucidate their formation thoroughly. The above-mentioned compounds belong to the class of polyketides and are built by multi-enzyme polyketide synthases (PKS) via repetitive catalytic cycles of linking elongation units to a starter molecule [25,26]. In regard to fungal PKSs, a differentiation between reducing (R-PKS) and non-reducing PKS (NR-PKS) is made based on extent of reductive processing and domain composition [27]. For R-PKS, the conserved structure from N- to C-termini consists of keto synthase (KS), acyl transferase (AT), dehydratase (DH), C-methyl transferase (C-MeT), enoylreductase (ER), ketoreductase (KR), and acyl carrier protein (ACP) domains [27,28]. In comparison, NR-PKS contain starter unit:acyl-carrier protein transferases (SAT) and a product template (PT) domain, which are unique among the PKS families [26,29]. The former connects the chain initiating compound to the enzyme, whereas the latter regulates cyclization reactions of the highly reactive and completely elongated intermediate to specific aromatic compounds [26,29,30,31]. Organization of most NR-PKS domains was found to be SAT-KS-AT-PT-ACP-ACP-TE [32].

For further investigation of secondary metabolite production, the underlying mechanisms need to be elaborated. Through advancement in the fields of next generation sequencing (NGS), it became possible to locate potentially involved genes. When several genes participating in formation of a secondary metabolite are located in close vicinity rather than spread over the genome, the term biosynthetic gene cluster (BGC) is applied [33,34,35,36,37]. Recent studies have identified BGCs from lichenized fungi responsible for the biosynthesis of secondary metabolites, including those for pigments, terpenes, and polyketides [38,39,40,41,42].

In order to identify specific BGCs, it is necessary to have access to the molecular makeup of the symbionts. This can be achieved by simultaneously sequencing the complete metagenome, which provides a comprehensive view of the BGCs of all organisms involved in the lichen association. By doing so, potential interactions between multi-species symbioses could be detected at the level of BGCs [4]. In addition, genetic accessibility is enabled to symbiotic partners which are not or challenging to culture [43]. These clusters are often located on the genome of the fungal partner of the lichen [34] and are activated in response to environmental cues [44,45]. However, one BGC may encode for various structurally familiar compounds [46,47,48]. Sequencing a pool of microorganisms using short read sequencing methods can be challenging, as nucleotide variances among different species can be inadequately represented or lost during the metagenomics analysis. This is also true for complex regions, making it difficult to efficiently assemble the complete genomes of all species in the sample [49,50,51]. Long read sequencing techniques instead, are able to provide a more comprehensive view of the genome of different microorganisms within the metagenome sample, thus resulting in high quality data output [52]. Furthermore, the long reads can be used to detect nucleotide variances among symbionts [53]. For the first time, the BGCs of the complete holobiont-especially algae and bacteria- directly from lichen thalli were identified.

In this study, we present two new lichen metagenomes of the genus *Hypogymnia, Hypogymnia physodes* (HPH) and *H. tubulosa* (HTU). *H. physodes* is one of the most ubiquitous and abundant macrolichens throughout Europe, and also occurs in North America, Africa, and Asia. It is a foliose, polymorphic species, up to 10 cm diameter in size, and forms lip-shaped soralia at the tips of the lobes. It is found on acidic substrata, including the bark of trees, and siliceous rocks. *H. tubulosa* is similar to *H. physodes*, but the lobes are more tubular, and soralia rounded, capitate, not lip-shaped. It has a similar distribution and grows on similar substrata as *H. physodes*, but is less frequent [54,55]. Both species often grow together, and both are associated with green algae of the genus *Trebouxia*. Natural products found in *Hypogymnia* are atranorin, chloroatranorin; physodic acid, 3-hydroxyphysodic acid, physodalic acid; and occasionally protocetraric, fumarprotocetraric, and usnic acid, some of which are medicinally relevant [56,57,58]. Consequently, these metagenomes provide an important resource for further research on the biosynthetic potential of the genus *Hypogymnia*.

## 2. Materials and Methods

### 2.1. Lichen Sample Collection

Samples were collected in Germany, Bavaria, Altenschneeberg (August 2022), from the bark of conifers, where they grew side by side. Precise locations of sequenced samples are listed in Table 1. To ensure correct lichen identity, a BLAST search on ITS sequences was performed. Lichen samples included in this study were identified as HPH and HTU.

### 2.2. GC-MS Analysis of Lichen Compounds

In addition, parts of the collected samples were analyzed by GC-MS to investigate the secondary metabolite composition. A measure of 500 mg of dry lichen biomass was macerated in 10 mL methanol for 24 h at 300 rpm. The obtained extract was analyzed by a Trace GC-MS Ultra system with DSQII (Thermo Scientific, Waltham, MA, USA). A TriPlus autosampler was employed to inject 1 µL of sample volume in split mode onto a SGE BPX5 column (30 m, I.D 0.25 mm, film 0.25 µm); injector temperature was set to 280 °C. Initial oven temperature was kept at 50 °C for 2.5 min. The temperature was increased with a ramp rate of 10 °C/min to 320 °C with a final hold step for 3 min. The carrier gas in this study was helium with a flow rate of 0.8 mL/min and a split ratio of 8. The mass spectra and chromatograms were recorded at 70 eV (EI). Detection of masses took place between 50 *m*/*z* and 650 *m*/*z* in the positive mode [53]. Compounds were identified by spectral comparison with a NIST/EPA/NIH MS library version 2.0.

### 2.3. High Molecular Weight DNA (HMW gDNA) Extraction and Library Preparation

Prior to DNA extraction, the respective lichen thallus was investigated under a binocular microscope to remove moss, wood, and other lichens from the sample. In addition, parts of the thallus, which were visibly infected by parasitic fungi were removed to minimize contaminants in the sample.

HMW gDNA extraction was conducted as follows. Lichen HMW gDNA was extracted using the Quick-DNA Fungal/Bacterial Miniprep Kit (Zymo Research, Europe GmbH, Freiburg, Germany). Dry thallus material of *Hypogymnia* samples was ground to fine powder in liquid nitrogen. The homogenized material was transferred into Bashing Bead Buffer of the kit. Genomic HMW DNA was isolated according to the manufacturer’s instructions. Due to the high content of polysaccharides, phenolic compounds and pigments further, purifications were necessary and carried out using the Genomic DNA Clean and Concentrator-10 Kit (Zymo Research, Europe GmbH, Freiburg, Germany) and the DNeasy PowerClean Clean up Kit (Qiagen, Venlo, The Netherlands). The quality of obtained HMW gDNAs was assessed with Nanophotometer (Implen, Munich, Germany), Qubit 2.0 Fluorometer (Thermo Scientific, Waltham, MA, USA) and TapeStation (Agilent Technologies, Santa Clara, CA, USA). SMRT bell libraries were constructed for samples passing the quality control (260/280 absorbance ratio of 1.75–1.85 and a 260/230 absorbance ratio of 2.0–2.2) according to the instructions for the Low DNA Input Protocol of the SMRT bell Express Prep kit v2. (Pacific Bioscience, Menlo Park, CA, USA).

Total input DNA (size: 10–18 kb) for the library generation was approximately 350–600 ng. The ligation with T-overhang SMRT bell adapters was performed at 20 °C for 1 h. After two clean up steps with AMPure PB beads, the size and concentration of the final libraries were assessed using TapeStation and Qubit Fluorometer 2.0 with Qubit dsDNA HS reagents Assay Kit.

### 2.4. Genome Sequencing

Whole genome sequencing of prepared lichen HMW gDNA libraries was conducted with a PacBio Sequel Iie device (Pacific Bioscience, Menlo Park, CA, USA). Pre-extension and adaptive loading (target of p1 + p2 = 0.95) were set to two hours with an on-plate concentration of 90 pM. The movie time was set to 30 h [59].

### 2.5. RNA Long Read Isoseq and Illumina Short Read Sequencing

In this study, long read IsoSeq (Pacific Bioscience, Menlo Park, CA, USA) and short read Illumina (NovaSeq, Illumina, San Diego, CA, USA) RNA sequencing were performed. To increase the quality of the genome assembly, transcripts were sequenced to add more depth and accuracy to the proposed gene models. For RNA extraction, frozen, unthawed lichen thalli were grinded using a CryoMill (Retsch, Haan, Germany) and a RNeasy Plant Mini Kit (Qiagen, Venlo, The Netherlands). To further clean the obtained RNA, a Turbo DNA free Kit (Invitrogen) was used. For long read RNA sequencing, an IsoSeq library preparation was performed on high quality RNA using the Sequel II Binding Kit 3.2 and SMRTbell prep kit 3.0. (Pacific Bioscience, Menlo Park, CA, USA). Transcriptome sequencing on Sequel IIe was performed at 24 h of movie time with an on-plate concentration of 80 pM. In case of short read RNA sequencing, samples were processed on a NovaSeq device, employing a paired-end run mode and 2 × 150 bp read length. Therefore, total RNAs were extracted with TRI Reagent (Zymo Research, Europe GmbH, Freiburg, Germany) according to the manufacturer’s instructions. Further purification was obtained by processing the samples with the RNA Clean and Concentrator-5 Kit (Zymo Research, Europe GmbH, Freiburg, Germany). This step was repeated until a 260/280 absorbance ratio of 1.9–2.1 and a 260/230 absorbance ratio of 1.8–2.2 were obtained. Only RNAs with a RIN value >8.0 (TapeStation) were used for sequencing.

### 2.6. Bioinformatic and Statistical Analysis

Metagenomic reads generated from whole lichen thalli predominantly contain fungal sequences from the mycobiont [60], which may pose problems to genome assemblers using solid *k*-mers. In this case, highly prevalent species will be over-represented and low-abundance species such as the photobiont will fail to assemble. To circumvent these issues, obtained Sequel IIe CCS long reads were assembled using metaFlye v2.9.1, as it is suitable to process read coverages of high non-uniformity. In addition, a simultaneous scaffolding of the assembled contigs was performed with flye, enabling further bioinformatic processing [61].

To distinguish obtained datasets, a taxonomical binning was conducted deploying blastx in the DIAMOND v2.0.14 algorithm [62] on a custom made database and MEGAN6 LR Community Edition v6.21.7 pipeline [63]. The DIAMOND database contained protein sequences of the following taxonomic groups: fungi, bacteria, archaea, viruses, chlorophyta, klebsormidiophyceae, tremella, and cystobasidium. For DIAMOND, the flags--*more-sensitive --frameshift 15* and --*rage-culling* were employed to account for insertion and deletion errors in long read sequencing with a frame-shift-aware alignment mode [64]. Resulting files were further processed in MEGAN by matching taxonomically assigned sequences to respective bins [65]. Consequently, contigs and scaffolds of desired nodes were extracted and subjected to consecutive analysis. To assess genome completeness and quality of resulting bins for further investigation, BUSCO, v5.3.2 (Benchmarking Universal Single-Copy Orthologs) [66], QUAST, v5.2.0 (Quality Assessment Tool) [67] and SeqKit v2.3.1 [68] were applied. For the validation of mycobiont and photobiont identities and distribution in investigated bins, an ITSx v1.1.3 analysis was performed [69]. Gene prediction with AUGUSTUS v3.4.0/BRAKER v2.1.6 is conducted, based on metagenomics data and respective transcriptomic data as hints [70,71,72]. This enables the functional annotation of genes in the respective datasets. A consecutive annotation of BGCs present in the obtained bins is performed by antiSMASH v6.1.1 [73], whereas Cluster of Orthologous Groups (COG) and Gene Ontology (GO) terms are levied by the EggNOG Mapper (v2.1.5) [74]. AntiSMASH, EggNOG, ITSx, and SeqKit analyses were performed on the Galaxy servers [75].

## 3. Results and Discussion

### 3.1. Volatile Compound Identification

**Figure 1:**
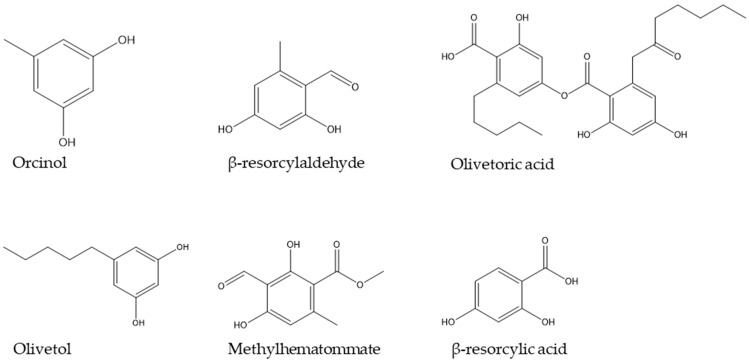
Molecule structures of lichen compounds identified by GC-MS.

GC-MS was used to identify volatile compounds in the macerated lichen samples. As shown in literature, lichen contain a plethora of secondary metabolites varying from depsides, depsidones, to dibenzofurans over to resorcylic-like compounds and anthraquinones [17]. In Figure 1, the polyketide-related compounds for both lichen samples are depicted. These are based on GC-MS data, which can be found in Table S1. Results showed similar compounds for both samples. These complex structures harbor aromatic ring elements as well as aliphatic elements and different chiral centers. In total chemical synthesis, complex biological molecules are not produced in high yields due to these centers and also to chemically reactive substituents as seen for the total synthesis of tetracycline [76]. Thus, a biological production system would be favorable. In nature, complex molecules are mostly secondary metabolites as part of multi-enzyme biosynthesis pathways [77]. The connected enzymes can be found on distinct clusters. Therefore, it is necessary to provide a comprehensive and high-quality genome to enable accessibility of the production-related genes.

### 3.2. Genome Sequencing and Quality Assessment

To present sequencing quality, the sequencing metrics of the two lichen samples HPH and HTU are listed in Table 2. Obtained metrics are comparable in each sample with only slight deviations. Due to mean HiFi Read Quality well above Q20, all samples were eligible for further bioinformatic analyses.

After taxonomic evaluation of both lichen samples, bins on family and genus level representing the different taxonomic groups of the investigated *Hypogymnia* holobionts were selected, in order to provide a sufficient insight on community composition. Table 3 shows the chosen bins for HPH and HTU divided into eukaryotic and bacterial origin. The identified fungi belonging to *Herpotrichiellaceae* and *Pleosporineae* are lichen-associated Ascomycota which possibly belong to the so-called “black fungi” (*Eurotiomycetes* and *Dothideomycetes*) [78].

To evaluate genome completeness and reliability for further data processing, a BUSCO [66] assessment was performed on the respective bins of the lichen metagenome. In both samples, the bin “*Parmeliaceae*” included sequences of the mycobiont and the bin “*Trebouxia*” sequences of the photobiont. Unfortunately, the amount of lichen genomes available in databases is too low to provide a selection on lower taxonomic level. Bin compositions mainly deviated in regard to *Herpotrichiellaceae*, which was not prominent in HTU, whereas the bin of *Verrucomicrobiota* was not as prevalent in HPH as the other investigated bins.

**Figure 2:**
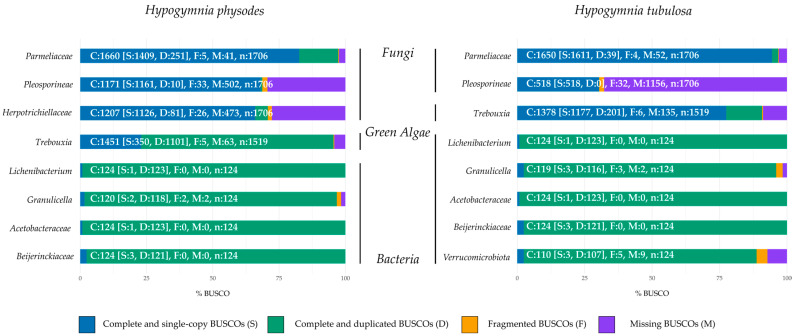
BUSCO genome completeness assessment of the *Hypogymnia physodes* (HPH) and *Hypogymnia tubulosa* (HTU) holobiont. Lineage datasets utilized to assess completeness: *Ascomycota*, *Chlorophyta*, and *Bacteria*.

Subsequently, the metagenomic bins were compared to the respective BUSCO gene sets of *Ascomycota*, *Chlorophyta* and *Bacteria*. These taxonomic groups were selected to provide a reliable insight in regard to genome representation of the lichen community. Obtained BUSCO results are shown in percentages to allow for an accurate comparison between the investigated bins. Figure 2 depicts the normalized and summarized results for each lichen sample by respective bin. All columns also include the absolute numbers to allow for more precise comparison between different bins.

In order to evaluate genome completeness of the mycobiont and other fungal bins, the orthologous gene sets of the phylum *Ascomycota* odb10 were chosen. In HPH, the fungal bins contained predominantly complete and single BUSCOs (66–82.6%) with the highest values in *Parmeliaceae*. Duplicated genes were observed in all fungal bins ranging from 0.6 to 14.7%, increasing from *Pleosporineae* over *Herpotrichiellaceae* to *Parmeliaceae*. These findings are based on fungal diversity in lichen, as the presence of different fungal genomes results in multiple detections of investigated gene sets by BUSCO [3]. In regard to missing BUSCO gene sets, approximately 29% were absent in *Herpotrichiellaceae* and *Pleosporineae*. As the primary mycobiont makes up most of the lichen biomass, this genome is predominately sequenced compared to other fungi included in the metagenome. Fragmentation on BUSCO gene sets ranged from 0.3 to 1.9%.

The photobiont (*Trebouxia*) was compared to the gene set of *Chlorophyta odb10*, which yielded mainly complete and duplicated BUSCOs (72.5%), with complete and single BUSCOs making up the majority of the remaining gene sets (23%). These findings are in line with the previously elaborated influence of biomass distribution in lichens on sequencing depth. The share of missing BUSCOs was about 4%, whereas 0.3% were fragmented.

BUSCO evaluation of the bacterial factions of the investigated lichen community against the gene set *Bacteria* yielded high duplication rates in every bin (95–99.2%). As the investigated bins encased more than one bacterial species, duplication rates were inevitable [79]. By investigating the genome completeness of the fungal bins present in HTU, the *Parmeliaceae* bin exhibits mainly complete and single BUSCOs with only slight duplication or fragmentation. In contrast, a high rate of missing BUSCOs was seen in the bin containing *Pleosporineae*. The node of this bin was smaller in comparison to the one in HPH (see Table S2). These findings may also be based on the share of primary mycobiont present in the lichen sample.

Comparable results are observed in the photobiont bin. Here the duplication rate is considerably lower than in the respective bin of HPH. These findings may suggest a uniform composition of algal partners in this lichen sample and a more diverse composition of algal strains in HPH. The bacterial faction of HTU displayed comparable results to HPH regarding duplication rates, based on the same rationale. The assessment of gene set completeness showed a similar pattern to that of genome completeness across all taxonomic groups belonging to eukaryota (Supplementary Figure S1). For bacterial bins, more complete and singe BUSCOs were observed by analysis of gene set completeness. However, the rate of missing and fragmented BUSCOs increased.

A second approach to evaluate the contiguity of the assembled metagenomes involves the utilization of the quality assessment tool for genome assemblies (QUAST). Thus, the different bins were assessed based on their genome size, number of contigs, and N50 values; a summary of the statistics for each bin are provided in Table 4 and Table 5. Notably, the N50 values for the primary symbionts, *Parmeliacea* sp. and *Trebouxia* sp., were around 1 Mb for both lichen samples. These findings, combined with the low L50 value for each, suggest with a high degree of confidence, that the genomes are assembled contiguously. A 1.4-fold difference in total contig length of *Parmeliacea* is observed between HPH and HTU, which could be due to natural variations in genome size. However, both bins align with the average size of the assembled *Parmelia* spp. genome reported in the literature (45 Mb, NCBI BioSample: SAMN17391792). A 1.6-fold difference in genome size can be seen in the *Trebouxia* bins; here, it is worth mentioning that in HTU, the missing BUSCOs differ by a factor of two. This may cause variations in observed total contig length. Deviation from the 69.35 Mb genome size (NCBI BioSample: SAMD00066476 [80]) reported in the literature may be explained by the nature of the *Trebouxia* bin, as it possibly encloses more than one algal partner in HPH [81].

The bacterial bins in HPH and HTU exhibit a high number of contigs of up to 2647. However, it should be noted that the reported genome sizes for *Lichenibacterium*, *Acetobacteraceae*, and *Granulicella* are around 6 Mb (NCBI BioSample: SAMN09781801), 3 Mb (NCBI BioSample: SAMN29020633), and 6 Mb (NCBI BioSample: SAMN28407668, [82]), respectively. Usually, bacterial genomes processed by long read sequencers are expected to be one circular contig only. This suggests that multiple organisms belonging to these genera may be present in a single bin. Additionally, the high duplication rates observed in all bacterial bins by the above BUSCO data is consistent with the high number of contigs and contig lengths found. By investigating the average coverage in both lichen samples, a strong bias towards the mycobiont is observed, 251- and 284-fold coverage in HPH and HTU, respectively. These findings are in line with the aforementioned distribution of mycobiont biomass in lichen. The algal and other fungal partners in both samples are comparable in coverage. As the bacterial bins of HPH and HTU differed in size, it was also expected to be observed in regard to coverage.

Comparison of GC content yields similar results throughout all compared taxonomic groups in both lichen samples.

### 3.3. Gene Models and Functional Annotation

Annotation of gene models was conducted with AUGUSTUS, utilizing the presented metagenome assemblies and a long-read IsoSeq database functioning as hints [70,71,72]. Here transcriptomic data from each lichen sample was deployed as an individual training set to ensure correct gene predictions for all factions of the respective metagenome [83]. For both lichen samples, the predicted genes resembling putative proteins were summed up from all encased bins investigated in this study. This yielded a total gene count of 133,963 for HPH and 96,257 for HTU. In addition, statistics on average genes length, gene density, and introns per gene were evaluated to further validate obtained gene predictions. Exact numbers for each bin are depicted in Table 6.

To further elaborate the metagenomes, a functional annotation was conducted based on the databases of Cluster of Orthologous Groups (COG) and Gene Ontology (GO) terms. Results were similar in both lichen samples and their respective bins. Annotation rates differed from ~85% in *Parmeliaceae* to ~94% in *Trebouxia* regarding the eukaryotic fraction, whereas approximately ~93% in the bacterial bins could be annotated. Interestingly, the bins of *Parmeliaceae* and *Trebouxia* harbored ~26% and ~20% of predicted genes with no assigned function, making further research on these organisms highly interesting.

**Figure 3:**
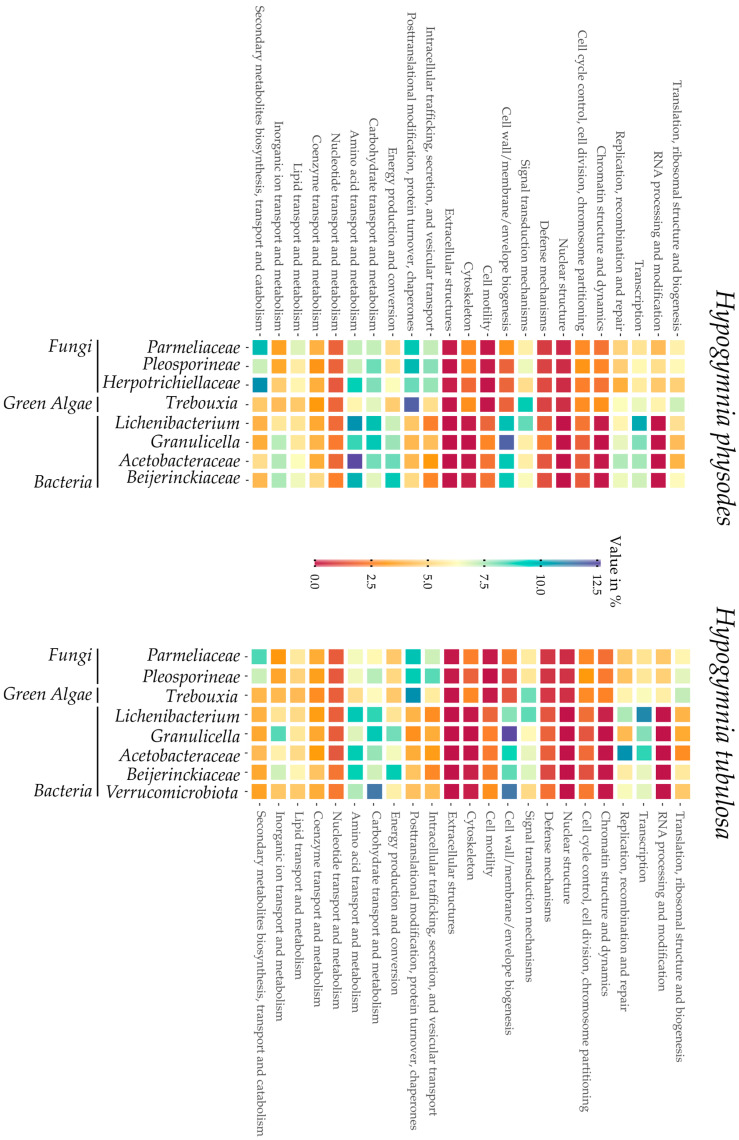
Heatmaps summarizing Cluster of Orthologous Groups (COG) from all bins of both lichen samples *Hypogymnia physodes* (HPH) and *Hypogymnia tubulosa* (HTU). Bins are grouped by taxonomic group in the following order: fungi, green algae, and bacteria.

Visualization of the COG distribution in the investigated lichen samples by each bin is depicted in Figure 3. From left to right, the bins are sorted by taxonomic group, beginning with the fungal fraction of the lichen metagenome over the green algae and ending with the bacterial bins. This overview provides insights into the respective functionalities of the predicted genes in each bin. Furthermore, strong differences between eukaryotes and prokaryotes are highlighted by a deep red or blue coloring in e.g., chromatin structure or cell wall biogenesis. On first sight, both heatmaps display a comparable coloring in the respective bins, thus highlighting the high similarity of the samples HPH and HTU. In regard to biosynthetic potential of the *Hypogymnia* holobiont, the row dedicated to “secondary metabolite biosynthesis” is elaborated further. Intriguingly, both *Parmeliaceae* bins possess high amounts of proteins predicted to be involved in secondary metabolite production. Other fungal bins equally displayed increased values compared to the bacterial faction or the photobiont in the same category. These findings are coherent with the observation of enhanced secondary metabolite production in lichen mycobionts and fungi [17,84,85,86,87].

### 3.4. Biosynthetic Gene Clusters

**Figure 4:**
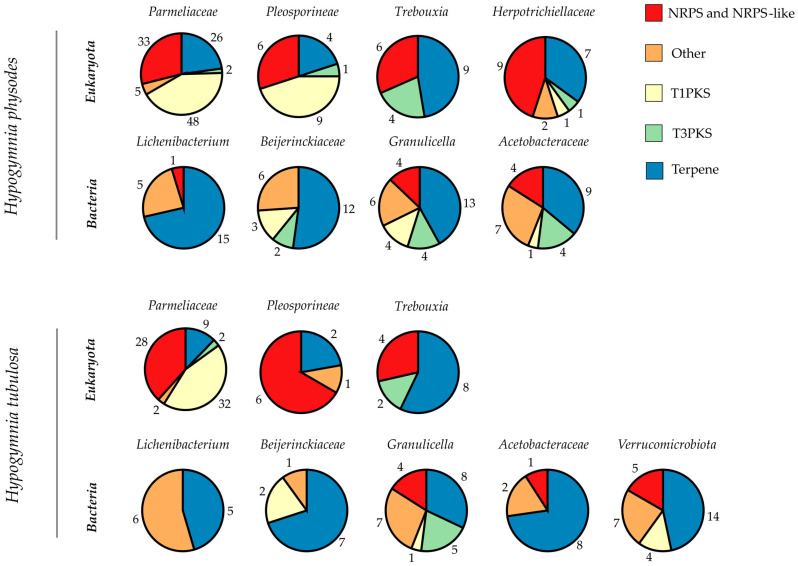
Biosynthetic Gene Cluster (BGC) analysis of *Hypogymnia physodes* (HPH) and *Hypogymnia tubulosa* (HTU), results by antiSMASH 6.1.1. Each bin was analyzed separately in order to give an overview of BGCs in different lichen fractions for both samples. Bins are grouped by the corresponding lichen. The fractions of the pie charts are computed based on the total number of BGCs contained in that bin. Numbers next to each fraction reflect total numbers of the particular BGC type present. The different BGC types are depicted by the color code on the top right.

Since the genus *Hypogymnia* is known to produce a manifold secondary metabolite spectrum, a deeper investigation into the biosynthetic pathways may yield intriguing results [8,88,89]. Therefore, the here-presented metagenomes were evaluated for biosynthetic gene clusters (BGCs) by antiSMASH 6.1.1 fungal/bacterial version [73], based on the previously conducted gene annotation. Furthermore, both *Trebouxia* bins were annotated by the fungal version of antiSMASH because plantiSMASH yielded insufficient results as previously described [90]. With this in silico approach, the biosynthetic enzymes involved in the formation of unique lichen substances are rendered accessible for wet lab research. To visualize the biosynthetic potential of the genus *Hypogymnia*, each bin’s BGC composition was categorized by putative function (Figure 4). Bins are grouped according to lichen sample and subdivided by superkingdom. The pie charts were computed based on total numbers of BGCs in the respective bin, including numbers representing the amount of BGCs of the various types. Main BGC categories identified by antiSMASH were nonribosomal peptide synthetases (NRPS and NRPS-like), type I and III polyketide synthases (T1PKS, T3PKS), and terpenes. All remaining types were grouped as “Other”. The complete list of all identified BGCs is given in Tables S3 and S4.

Most BGCs annotated throughout both lichen samples were related to terpene formation, followed by decreasing numbers of NRPS over T1PKS and ending with T3PKS. To allow for a direct comparison, the total numbers of BGCs per bin are listed in Table 7. For the first time, the photobiont and the bacterial factions were investigated directly from a lichen thallus. This gives information on the BGC composition of the complete holobiont as found in nature.

The prokaryotic faction in HPH exhibited an average of 26 BGCs over all four bins. For HTU, an average of ~17 BGCs was reported. Some of the bacterial bins exhibit BGC totals in the ranges comparable to *Streptomyces* strains, which are known for their multitude of BGCs (25–70) [91]. However, as the bins contain more than one bacterial genome the exact numbers of BGCs may differ between individual species. Main BGC class assigned by antiSMASH was related to terpene formation. Throughout all bacterial bins, only 26 BGCs were with assigned function. The influence of lichen-associated bacteria on the symbiosis still remains mostly unclear [92]. Identification of bacterial BGCs may yield insights on further functions in addition to the already known provision of vitamins and cofactors used for degradation of phenolic compounds [93]. As most of the BGCs remain without assigned function, these may be intriguing for further research.

A closer look into the photobiont bins yields a comparable distribution of BGCs in the respective categories, even if the BUSCO and QUAST results differed strongly (see Figure 2 and Table 4 and Table 5). For the algal bins, 19 (HPH) and 14 (HTU) BGCs were annotated which exceeds the numbers reported in previous studies [90]. This might be due to the nature of the investigated bins, enclosing more than one species. Therefore, an ITSx analysis was performed to elucidate the bin composition of *Trebouxia* and *Parmeliaceae*. For the bins in HPH only single ITS sequences were found, which is also true for the mycobiont bin in HTU. In regard to the photobiont bin in HTU, no ITS sequence was extractable. However, the absence of ITS sequences does not eliminate the possibility that other parts of the respective genome are present in the respective bins. This also applies to the findings of the BUSCO analysis. Results on ITSx are listed in Table S5. As algae from the genus *Trebouxia* exhibit slow growth rates or are often not yet amenable for cultivation [43], this metagenomic approach renders unidentified BGCs and genes accessible for genome mining. Intriguingly, antiSMASH yielded no assignment to any of the annotated BGCs, making these clusters interesting for further research. As the main class of photobiont BGCs is related to terpene production a formation of relevant natural products may be possible.

In comparison, the *Parmeliaceae* bins harbor the highest number of BGCs (114 in HPH; 73 in HTU) in the respective lichen. The richness in BGCs is in line with findings in previous studies concerning HTU, as the described BGC content in lichen ranges between 27 and 80 [10]. In the case of HPH, the total number of BGCs exceeded these amounts. Compared to other organisms harboring high amounts of BGCs such as *Streptomyces* spp. (23–80) [94,95], *Cyanobacteria* spp. (1–42) [96,97] *Myxobacteria* spp. (30–46) [98,99], and *Nocardia* spp. (~36) [100,101], the investigated *Parmeliaceae* bins showed comparable or higher totals of BGCs. For both mycobiont bins, the majority of BGCs were putative PKS followed by terpene related clusters. In regard to annotation by antiSMASH with the MIBiG database and Pfam, only 27 (HPH) and 16 (HTU) BGCs were with assigned function, highlighting the yet still untapped potential for further investigation.

## Figures and Tables

**Table 1:** Geographic coordinates of the lichen sample collection.

Lichen Species	Latitude	Longitude
*Hypogymnia physodes* (HPH)	49°26′14.3″ N	12°32′50.9″ E
*Hypogymnia tubulosa* (HTU)	49°26′14.3″ N	12°32′50.9″ E

**Table 2:** Metagenomic PacBio sequencing of *Hypogymnia physodes* (HPH) and *Hypogymnia tubulosa* (HTU).

Analysis Metrics	HPH	HTU
Total Bases (Gb)	678.17	656.10
HiFi Reads	3,584,910	3,461,456
HiFi Yield (Gb)	24.65	25.61
HiFi Read Length (mean, bp)	6877	7398
HiFi Read Quality (median)	Q42	Q43
HiFi Number of Passes (mean)	18	17

**Table 3:** Taxonomic binning of the lichen samples HPH and HTU.

Taxonomic Group	HPH	HTU
*Fungi*	*Parmeliaceae*	*Parmeliaceae*
	*Pleosporineae*	*Pleosporineae*
	*Herpotrichiellaceae*	*-*
*Green Algae*	*Trebouxia*	*Trebouxia*
*Bacteria*	*Lichenibacterium*	*Lichenibacterium*
	*Acetobacteraceae*	*Acetobacteraceae*
	*Granulicella*	*Granulicella*
	*Beijerinckiaceae*	*Beijerinckiaceae*
	*-*	*Verrucomicrobiota*

**Table 4:** QUAST results of *Hypogymnia physodes* (HPH) from all investigated bins. From left to right, bins were abbreviated as follows: *Parmeliaceae*, *Pleosporineae*, *Herpotrichiellaceae*, *Trebouxia*, *Lichenibacterium*, *Acetobacteraceae*, *Granulicella*, and *Beijerinckiaceae*.

Analysis Metrics	Parm	Pleo	Herpo	Treb	Licheni	Aceto	Granu	Beij
# contigs	697	614	992	497	1215	1807	1551	977
Largest contig	2,288,512	226,469	240,890	2,824,615	1,201,869	5,239,257	4,442,654	1,606,592
Total length	58,447,069	23,701,369	26,591,620	129,019,835	41,345,960	91,111,576	63,381,977	27,344,988
N50	1,017,384	50,386	34,766	1,073,013	80,917	152,096	78,893	51,614
L50	20	156	238	37	75	94	149	115
# N’s per 100 kbp	0.51	0	0	0.39	0	0.22	0	0.73
Average coverage	251.57	12.12	5.40	4.88	16.89	16.73	24.19	16.24
GC content	51.57	50.69	53.01	55.03	64.51	66.14	60.91	66.93

**Table 5:** Results from QUAST analysis in regard to selected bins from *Hypogymnia tubulosa* (HTU). From left to right, bins were abbreviated as follows: *Parmeliaceae, Pleosporineae, Trebouxia, Lichenibacterium, Acetobacteraceae, Granulicella, Beijerinckiaceae,* and *Verrucomicrobiota*.

Analysis Metrics	Parm	Pleo	Treb	Licheni	Aceto	Granu	Beij	Verruco
# contigs	102	385	726	1562	2647	602	685	708
Largest contig	3,149,693	100,164	4,890,443	1,108,507	2,374,220	4,366,399	1,094,751	4,601,209
Total length	39,875,705	10,235,779	76,898,651	56,755,182	107,354,954	35,634,928	20,508,045	33,036,627
N50	1,305,677	32,841	2,503,874	71,728	80,216	225,427	55,316	191,798
L50	11	108	12	149	276	19	69	27
# N’s per 100 kbp	0	0	0.13	0.35	0.28	0.28	0	0.3
Average coverage	284.19	15.85	3.77	32.01	24.34	75.31	15.71	13.92
GC content	51.75	51.35	55.22	64.64	66.60	59.14	66.40	61.18

**Table 6:** Gene prediction statistics on the taxonomic groups of HPH and HTU. The bins are abbreviated in decreasing order as follows: *Parmeliaceae*, *Pleosporineae*, *Herpotrichiellaceae*, *Trebouxia*, *Lichenibacterium*, *Acetobacteraceae*, *Granulicella*, *Beijerinckiaceae,* and *Verrucomicrobiota*. Average was abbreviated as “Av.”.

Bin	HPH				HTU			
	Gene Count	Av. Gene Length	Gene Density (Genes/Mb)	Introns/Gene	Gene Count	Av. Gene Length	Gene Density (Genes/Mb)	Introns/Gene
Parm	18,804	1455.3	307.48	2.72	13,242	1440.9	329.77	2.06
Pleo	7318	1575.3	385.67	1.75	3159	1870.8	307.74	2.88
Herpo	9247	1589.5	274.67	1.79	-	-	-	-
Treb	19,421	1641.00	148.22	7.33	13,458	1576.5	172.46	7.18
Licheni	13,225	681.4	319.89	-	11,950	649.9	210.57	-
Granu	19,260	931.8	303.87	-	11,423	861.9	221.00	-
Aceto	34,871	783.1	382.73	-	23,724	740.7	320.39	-
Beij	11,817	763.3	431.85	-	8303	709.7	404.87	-
Verruco	-	-	-	-	10,998	782.0	332.90	-

**Table 7:** Total numbers of BGCs per bin divided by sample.

	Parm	Pleo	Herpo	Treb	Licheni	Beij	Granu	Aceto	Verruco
BGCs HPH	114	20	20	19	21	23	36	25	/-
BGCs HTU	73	8	/-	14	11	10	25	11	30

## Data Availability

Data available in a publicly accessible repository. The data presented in this study are openly available in the National Center for Biotechnology Information (NCBI). BioSample accession number: *Hypogymnia physodes* SAMN34074577, *Hypogymnia tubulosa* SAMN34074619.

## Supplementary Materials

The following supporting information can be downloaded at: https://www.mdpi.com/article/10.3390/jof9050546/s1, Figure S1: BUSCO gene set completeness; Table S1: GC-MS spectra for HPH and HTU; Table S2: Bin comparison of both lichen samples; Table S3: Summary of antiSMASH HPH results on BGCs; Table S4: Summary of antiSMASH HTU results on BGCs; Table S5: ITSx analysis results.
